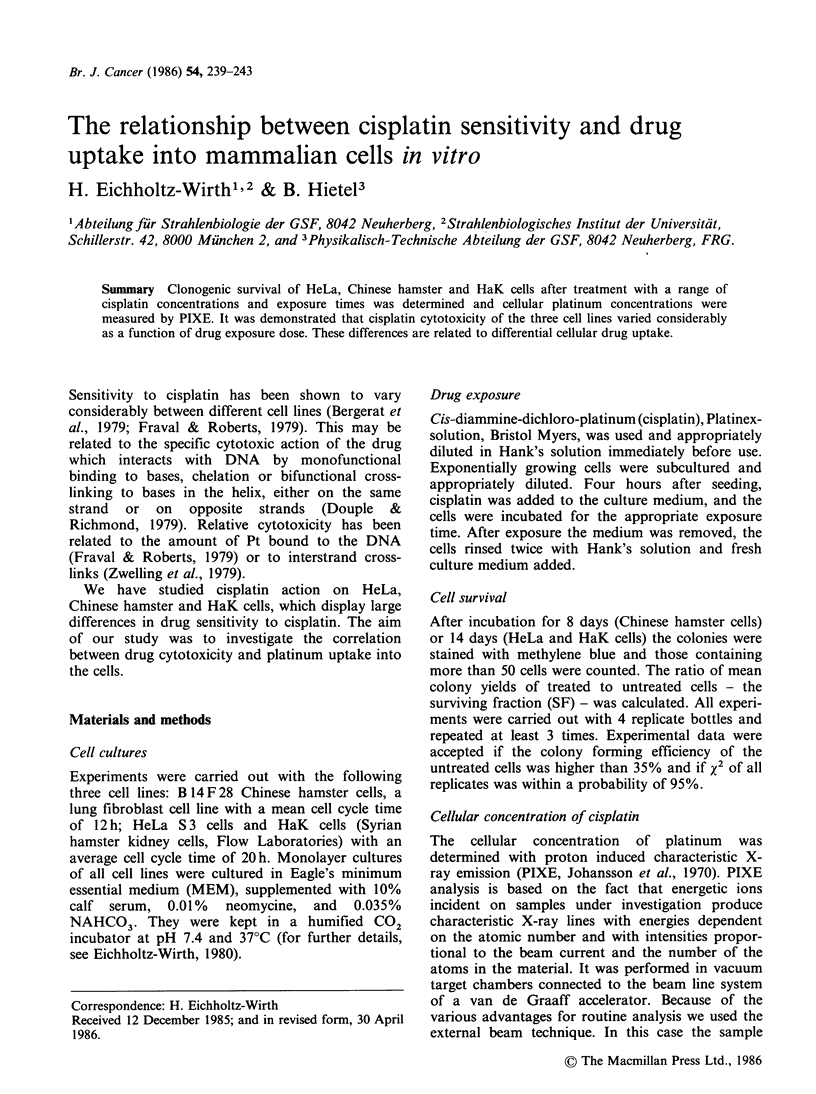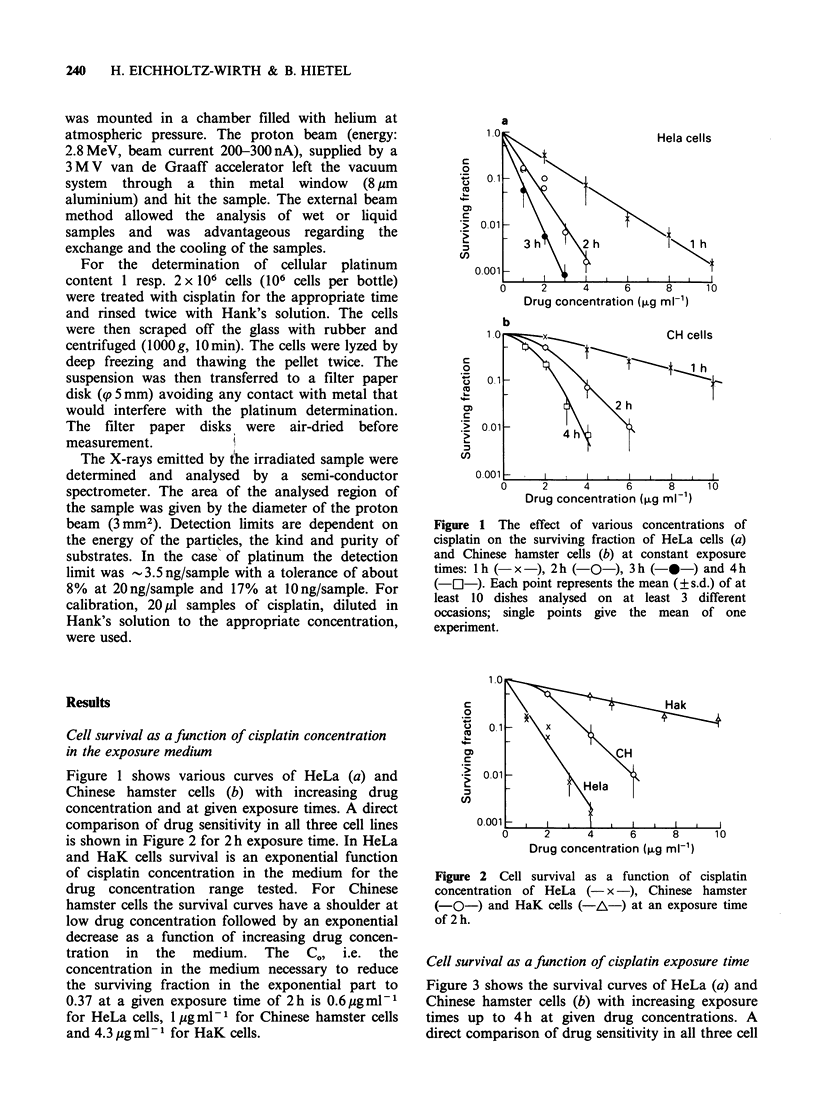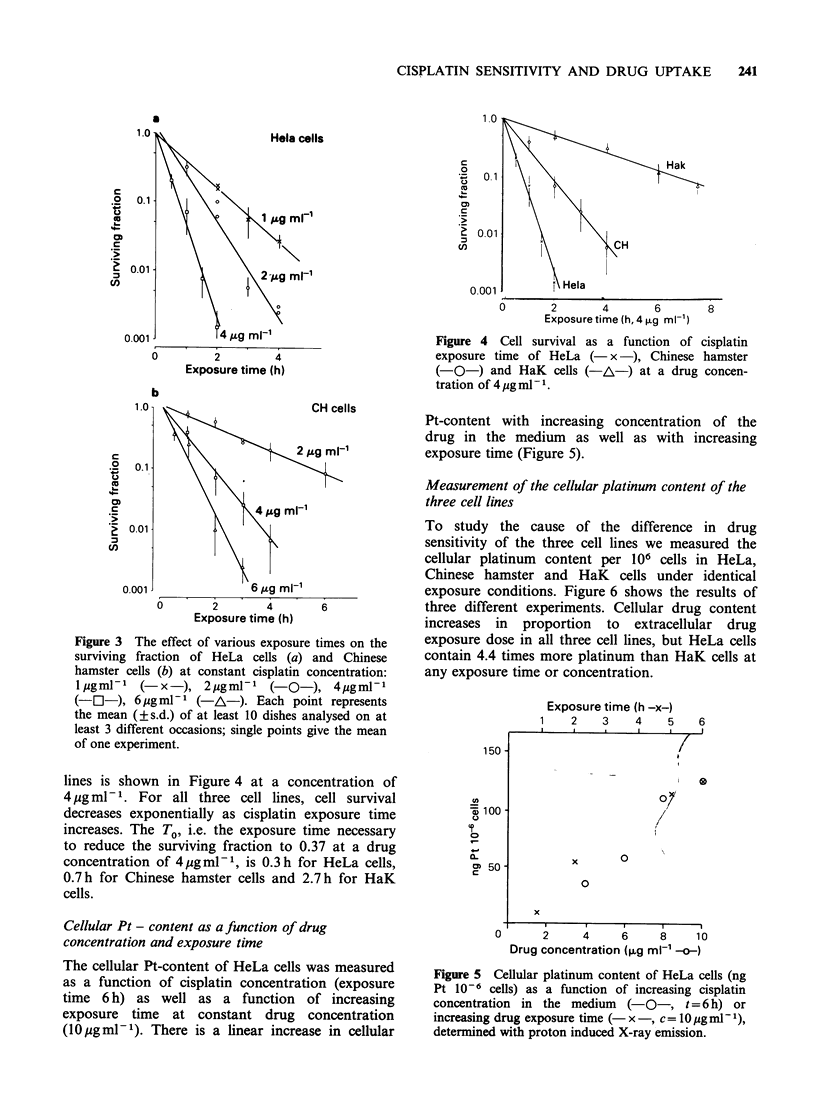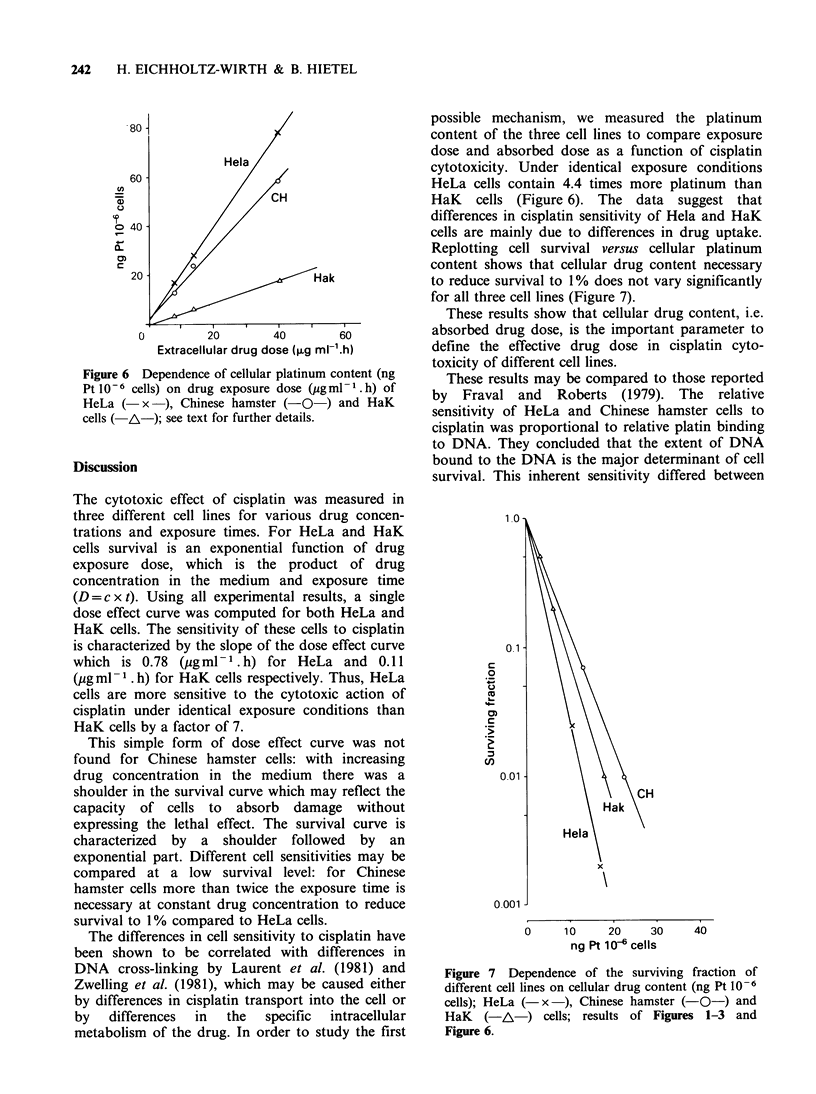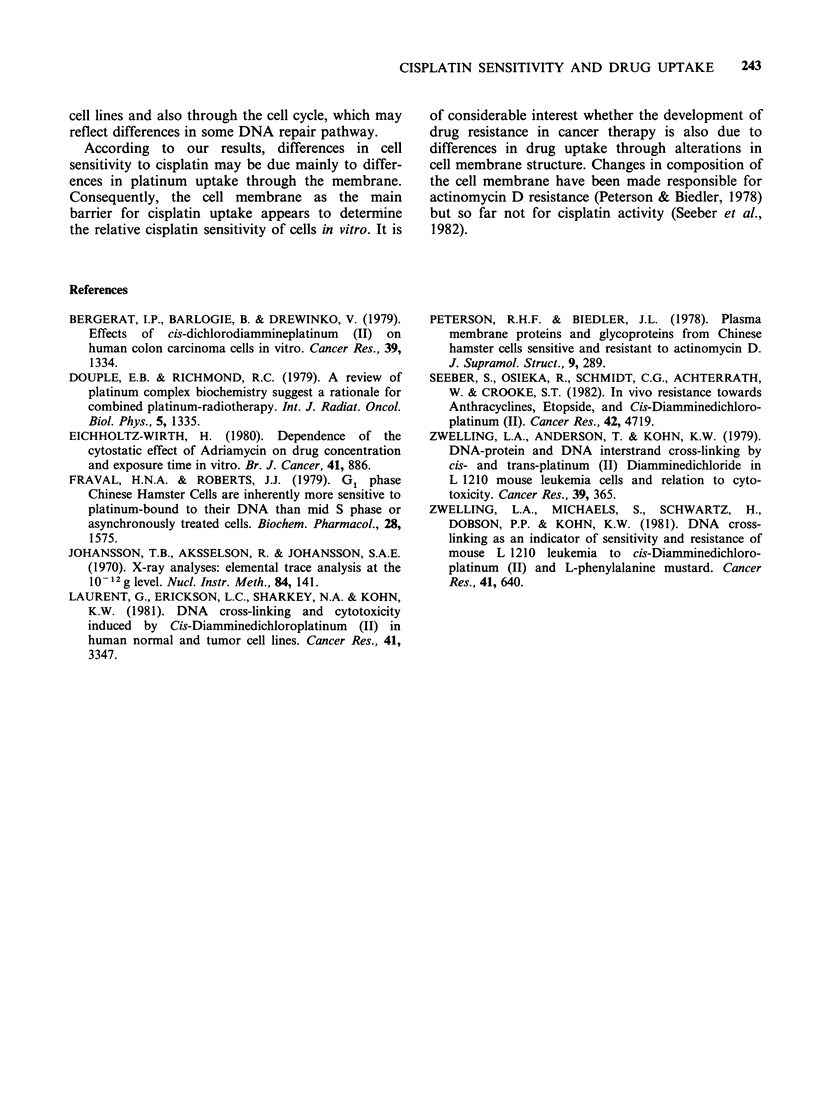# The relationship between cisplatin sensitivity and drug uptake into mammalian cells in vitro.

**DOI:** 10.1038/bjc.1986.168

**Published:** 1986-08

**Authors:** H. Eichholtz-Wirth, B. Hietel

## Abstract

Clonogenic survival of HeLa, Chinese hamster and HaK cells after treatment with a range of cisplatin concentrations and exposure times was determined and cellular platinum concentrations were measured by PIXE. It was demonstrated that cisplatin cytotoxicity of the three cell lines varied considerably as a function of drug exposure dose. These differences are related to differential cellular drug uptake.


Br. J. Cancer (1986) **54**, 239–243

### The relationship between cisplatin sensitivity and drug uptake into mammalian cells *in vitro*

H. Eichholtz-Wirth[1,2] & B. Hietel[3]

[1]*Abteilung für Strahlenbiologie der GSF, 8042 Neuherberg,* [2]*Strahlenbiologisches Institut der Universität, Schillerstr. 42, 8000 München 2, and* [3]*Physikalisch-Technische Abteilung der GSF, 8042 Neuherberg, FRG.*

**Summary** Clonogenic survival of HeLa, Chinese hamster and HaK cells after treatment with a range of cisplatin concentrations and exposure times was determined and cellular platinum concentrations were measured by PIXE. It was demonstrated that cisplatin cytotoxicity of the three cell lines varied considerably as a function of drug exposure dose. These differences are related to differential cellular drug uptake.

Sensitivity to cisplatin has been shown to vary considerably between different cell lines (Bergerat *et al.*, 1979; Fraval & Roberts, 1979). This may be related to the specific cytotoxic action of the drug which interacts with DNA by monofunctional binding to bases, chelation or bifunctional cross-linking to bases in the helix, either on the same strand or on opposite strands (Douple & Richmond, 1979). Relative cytotoxicity has been related to the amount of Pt bound to the DNA (Fraval & Roberts, 1979) or to interstrand cross-links (Zwelling *et al.*, 1979).

We have studied cisplatin action on HeLa, Chinese hamster and HaK cells, which display large differences in drug sensitivity to cisplatin. The aim of our study was to investigate the correlation between drug cytotoxicity and platinum uptake into the cells.

### Materials and methods

### *Cell cultures*

Experiments were carried out with the following three cell lines: B14F28 Chinese hamster cells, a lung fibroblast cell line with a mean cell cycle time of 12h; HeLa S3 cells and HaK cells (Syrian hamster kidney cells, Flow Laboratories) with an average cell cycle time of 20h. Monolayer cultures of all cell lines were cultured in Eagle's minimum essential medium (MEM), supplemented with 10% calf serum, 0.01% neomycine, and 0.035% $NAHCO_3$. They were kept in a humified $CO_2$ incubator at pH 7.4 and 37°C (for further details, see Eichholtz-Wirth, 1980).

### *Drug exposure*

*Cis*-diammine-dichloro-platinum (cisplatin), Platinex-solution, Bristol Myers, was used and appropriately diluted in Hank's solution immediately before use. Exponentially growing cells were subcultured and appropriately diluted. Four hours after seeding, cisplatin was added to the culture medium, and the cells were incubated for the appropriate exposure time. After exposure the medium was removed, the cells rinsed twice with Hank's solution and fresh culture medium added.

### *Cell survival*

After incubation for 8 days (Chinese hamster cells) or 14 days (HeLa and HaK cells) the colonies were stained with methylene blue and those containing more than 50 cells were counted. The ratio of mean colony yields of treated to untreated cells – the surviving fraction (SF) – was calculated. All experiments were carried out with 4 replicate bottles and repeated at least 3 times. Experimental data were accepted if the colony forming efficiency of the untreated cells was higher than 35% and if $\chi^2$ of all replicates was within a probability of 95%.

### *Cellular concentration of cisplatin*

The cellular concentration of platinum was determined with proton induced characteristic X-ray emission (PIXE, Johansson *et al.*, 1970). PIXE analysis is based on the fact that energetic ions incident on samples under investigation produce characteristic X-ray lines with energies dependent on the atomic number and with intensities proportional to the beam current and the number of the atoms in the material. It was performed in vacuum target chambers connected to the beam line system of a van de Graaff accelerator. Because of the various advantages for routine analysis we used the external beam technique. In this case the sample

Correspondence: H. Eichholtz-Wirth
Received 12 December 1985; and in revised form, 30 April 1986.

© The Macmillan Press Ltd., 1986

was mounted in a chamber filled with helium at atmospheric pressure. The proton beam (energy: 2.8 MeV, beam current 200–300 nA), supplied by a 3 MV van de Graaff accelerator left the vacuum system through a thin metal window (8 μm aluminium) and hit the sample. The external beam method allowed the analysis of wet or liquid samples and was advantageous regarding the exchange and the cooling of the samples.

For the determination of cellular platinum content 1 resp. $2 \times 10^6$ cells ($10^6$ cells per bottle) were treated with cisplatin for the appropriate time and rinsed twice with Hank's solution. The cells were then scraped off the glass with rubber and centrifuged (1000 *g*, 10 min). The cells were lyzed by deep freezing and thawing the pellet twice. The suspension was then transferred to a filter paper disk ($\varphi$ 5 mm) avoiding any contact with metal that would interfere with the platinum determination. The filter paper disks were air-dried before measurement.

The X-rays emitted by the irradiated sample were determined and analysed by a semi-conductor spectrometer. The area of the analysed region of the sample was given by the diameter of the proton beam (3 $mm^2$). Detection limits are dependent on the energy of the particles, the kind and purity of substrates. In the case of platinum the detection limit was ~3.5 ng/sample with a tolerance of about 8% at 20 ng/sample and 17% at 10 ng/sample. For calibration, 20 μl samples of cisplatin, diluted in Hank's solution to the appropriate concentration, were used.

**Figure 1** The effect of various concentrations of cisplatin on the surviving fraction of HeLa cells (*a*) and Chinese hamster cells (*b*) at constant exposure times: 1 h (—×—), 2 h (—○—), 3 h (—●—) and 4 h (—□—). Each point represents the mean (±s.d.) of at least 10 dishes analysed on at least 3 different occasions; single points give the mean of one experiment.

### Results

### *Cell survival as a function of cisplatin concentration in the exposure medium*

Figure 1 shows various curves of HeLa (*a*) and Chinese hamster cells (*b*) with increasing drug concentration and at given exposure times. A direct comparison of drug sensitivity in all three cell lines is shown in Figure 2 for 2 h exposure time. In HeLa and HaK cells survival is an exponential function of cisplatin concentration in the medium for the drug concentration range tested. For Chinese hamster cells the survival curves have a shoulder at low drug concentration followed by an exponential decrease as a function of increasing drug concentration in the medium. The $C_o$, i.e. the concentration in the medium necessary to reduce the surviving fraction in the exponential part to 0.37 at a given exposure time of 2 h is 0.6 μg $ml^{-1}$ for HeLa cells, 1 μg $ml^{-1}$ for Chinese hamster cells and 4.3 μg $ml^{-1}$ for HaK cells.

**Figure 2** Cell survival as a function of cisplatin concentration of HeLa (—×—), Chinese hamster (—○—) and HaK cells (—△—) at an exposure time of 2 h.

### *Cell survival as a function of cisplatin exposure time*

Figure 3 shows the survival curves of HeLa (*a*) and Chinese hamster cells (*b*) with increasing exposure times up to 4 h at given drug concentrations. A direct comparison of drug sensitivity in all three cell

**Figure 3** The effect of various exposure times on the surviving fraction of HeLa cells (*a*) and Chinese hamster cells (*b*) at constant cisplatin concentration: $1\mu g\,ml^{-1}$ (—×—), $2\mu g\,ml^{-1}$ (—O—), $4\mu g\,ml^{-1}$ (—□—), $6\mu g\,ml^{-1}$ (—△—). Each point represents the mean (±s.d.) of at least 10 dishes analysed on at least 3 different occasions; single points give the mean of one experiment.

lines is shown in Figure 4 at a concentration of $4\mu g\,ml^{-1}$. For all three cell lines, cell survival decreases exponentially as cisplatin exposure time increases. The $T_0$, i.e. the exposure time necessary to reduce the surviving fraction to 0.37 at a drug concentration of $4\mu g\,ml^{-1}$, is 0.3 h for HeLa cells, 0.7 h for Chinese hamster cells and 2.7 h for HaK cells.

*Cellular Pt – content as a function of drug concentration and exposure time*

The cellular Pt-content of HeLa cells was measured as a function of cisplatin concentration (exposure time 6 h) as well as a function of increasing exposure time at constant drug concentration ($10\mu g\,ml^{-1}$). There is a linear increase in cellular

**Figure 4** Cell survival as a function of cisplatin exposure time of HeLa (—×—), Chinese hamster (—O—) and HaK cells (—△—) at a drug concentration of $4\mu g\,ml^{-1}$.

Pt-content with increasing concentration of the drug in the medium as well as with increasing exposure time (Figure 5).

*Measurement of the cellular platinum content of the three cell lines*

To study the cause of the difference in drug sensitivity of the three cell lines we measured the cellular platinum content per $10^6$ cells in HeLa, Chinese hamster and HaK cells under identical exposure conditions. Figure 6 shows the results of three different experiments. Cellular drug content increases in proportion to extracellular drug exposure dose in all three cell lines, but HeLa cells contain 4.4 times more platinum than HaK cells at any exposure time or concentration.

**Figure 5** Cellular platinum content of HeLa cells (ng Pt $10^{-6}$ cells) as a function of increasing cisplatin concentration in the medium (—O—, $t$=6 h) or increasing drug exposure time (—×—, $c$=$10\mu g\,ml^{-1}$), determined with proton induced X-ray emission.

**Figure 6** Dependence of cellular platinum content (ng Pt $10^{-6}$ cells) on drug exposure dose ($\mu$g ml$^{-1}$.h) of HeLa (—×—), Chinese hamster (—O—) and HaK cells (—△—); see text for further details.

### Discussion

The cytotoxic effect of cisplatin was measured in three different cell lines for various drug concentrations and exposure times. For HeLa and HaK cells survival is an exponential function of drug exposure dose, which is the product of drug concentration in the medium and exposure time ($D = c \times t$). Using all experimental results, a single dose effect curve was computed for both HeLa and HaK cells. The sensitivity of these cells to cisplatin is characterized by the slope of the dose effect curve which is 0.78 ($\mu$g ml$^{-1}$.h) for HeLa and 0.11 ($\mu$g ml$^{-1}$.h) for HaK cells respectively. Thus, HeLa cells are more sensitive to the cytotoxic action of cisplatin under identical exposure conditions than HaK cells by a factor of 7.

This simple form of dose effect curve was not found for Chinese hamster cells: with increasing drug concentration in the medium there was a shoulder in the survival curve which may reflect the capacity of cells to absorb damage without expressing the lethal effect. The survival curve is characterized by a shoulder followed by an exponential part. Different cell sensitivities may be compared at a low survival level: for Chinese hamster cells more than twice the exposure time is necessary at constant drug concentration to reduce survival to 1% compared to HeLa cells.

The differences in cell sensitivity to cisplatin have been shown to be correlated with differences in DNA cross-linking by Laurent *et al.* (1981) and Zwelling *et al.* (1981), which may be caused either by differences in cisplatin transport into the cell or by differences in the specific intracellular metabolism of the drug. In order to study the first possible mechanism, we measured the platinum content of the three cell lines to compare exposure dose and absorbed dose as a function of cisplatin cytotoxicity. Under identical exposure conditions HeLa cells contain 4.4 times more platinum than HaK cells (Figure 6). The data suggest that differences in cisplatin sensitivity of Hela and HaK cells are mainly due to differences in drug uptake. Replotting cell survival *versus* cellular platinum content shows that cellular drug content necessary to reduce survival to 1% does not vary significantly for all three cell lines (Figure 7).

These results show that cellular drug content, i.e. absorbed drug dose, is the important parameter to define the effective drug dose in cisplatin cytotoxicity of different cell lines.

These results may be compared to those reported by Fraval and Roberts (1979). The relative sensitivity of HeLa and Chinese hamster cells to cisplatin was proportional to relative platin binding to DNA. They concluded that the extent of DNA bound to the DNA is the major determinant of cell survival. This inherent sensitivity differed between

**Figure 7** Dependence of the surviving fraction of different cell lines on cellular drug content (ng Pt $10^{-6}$ cells); HeLa (—×—), Chinese hamster (—O—) and HaK (—△—) cells; results of **Figures 1–3** and **Figure 6**.

cell lines and also through the cell cycle, which may reflect differences in some DNA repair pathway.

According to our results, differences in cell sensitivity to cisplatin may be due mainly to differences in platinum uptake through the membrane. Consequently, the cell membrane as the main barrier for cisplatin uptake appears to determine the relative cisplatin sensitivity of cells *in vitro*. It is of considerable interest whether the development of drug resistance in cancer therapy is also due to differences in drug uptake through alterations in cell membrane structure. Changes in composition of the cell membrane have been made responsible for actinomycin D resistance (Peterson & Biedler, 1978) but so far not for cisplatin activity (Seeber *et al.*, 1982).

### References

BERGERAT, I.P., BARLOGIE, B. & DREWINKO, V. (1979). Effects of *cis*-dichlorodiammineplatinum (II) on human colon carcinoma cells in vitro. *Cancer Res.*, **39**, 1334.

DOUPLE, E.B. & RICHMOND, R.C. (1979). A review of platinum complex biochemistry suggest a rationale for combined platinum-radiotherapy. *Int. J. Radiat. Oncol. Biol. Phys.*, **5**, 1335.

EICHHOLTZ-WIRTH, H. (1980). Dependence of the cytostatic effect of Adriamycin on drug concentration and exposure time in vitro. *Br. J. Cancer*, **41**, 886.

FRAVAL, H.N.A. & ROBERTS, J.J. (1979). $G_1$ phase Chinese Hamster Cells are inherently more sensitive to platinum-bound to their DNA than mid S phase or asynchronously treated cells. *Biochem. Pharmacol.*, **28**, 1575.

JOHANSSON, T.B., AKSSELSON, R. & JOHANSSON, S.A.E. (1970). X-ray analyses: elemental trace analysis at the $10^{-12}$ g level. *Nucl. Instr. Meth.*, **84**, 141.

LAURENT, G., ERICKSON, L.C., SHARKEY, N.A. & KOHN, K.W. (1981). DNA cross-linking and cytotoxicity induced by *Cis*-Diamminedichloroplatinum (II) in human normal and tumor cell lines. *Cancer Res.*, **41**, 3347.

PETERSON, R.H.F. & BIEDLER, J.L. (1978). Plasma membrane proteins and glycoproteins from Chinese hamster cells sensitive and resistant to actinomycin D. *J. Supramol. Struct.*, **9**, 289.

SEEBER, S., OSIEKA, R., SCHMIDT, C.G., ACHTERRATH, W. & CROOKE, S.T. (1982). In vivo resistance towards Anthracyclines, Etopside, and *Cis*-Diamminedichloroplatinum (II). *Cancer Res.*, **42**, 4719.

ZWELLING, L.A., ANDERSON, T. & KOHN, K.W. (1979). DNA-protein and DNA interstrand cross-linking by *cis*- and trans-platinum (II) Diamminedichloride in L 1210 mouse leukemia cells and relation to cytotoxicity. *Cancer Res.*, **39**, 365.

ZWELLING, L.A., MICHAELS, S., SCHWARTZ, H., DOBSON, P.P. & KOHN, K.W. (1981). DNA cross-linking as an indicator of sensitivity and resistance of mouse L 1210 leukemia to *cis*-Diamminedichloroplatinum (II) and L-phenylalanine mustard. *Cancer Res.*, **41**, 640.